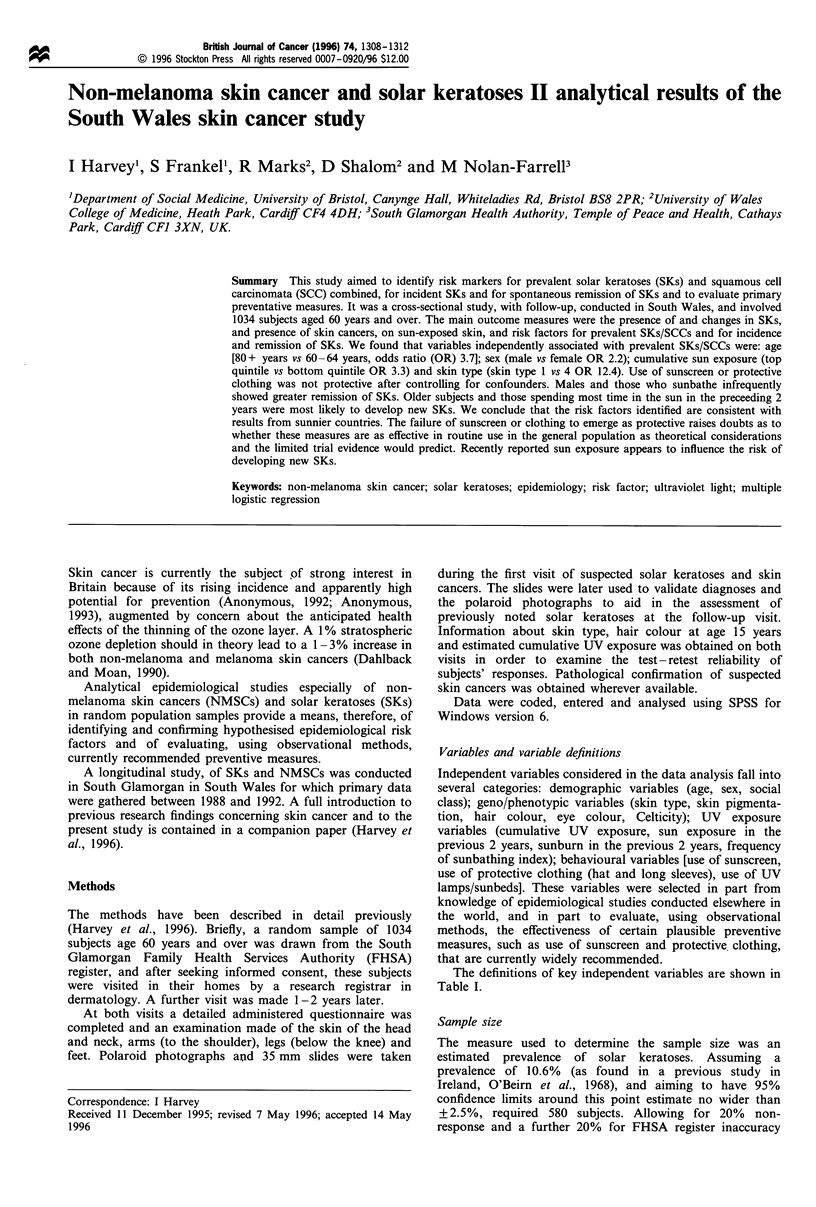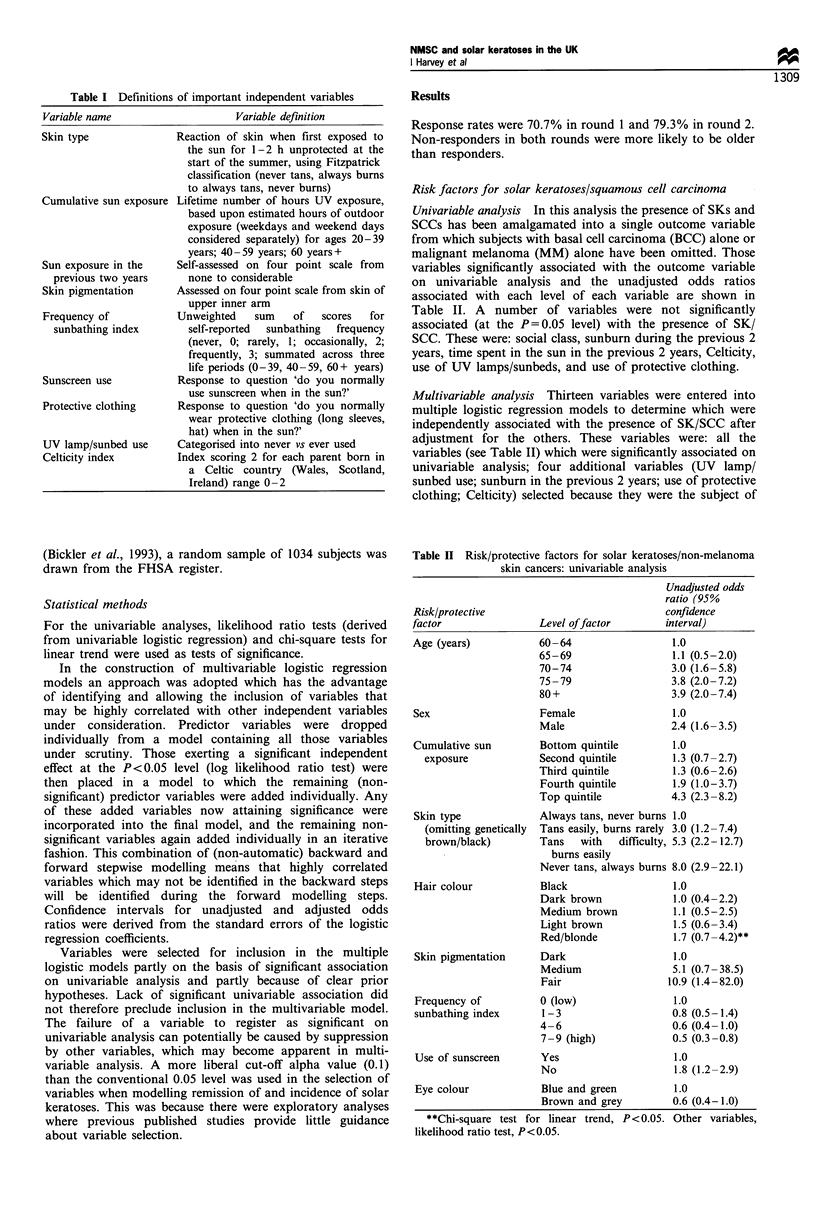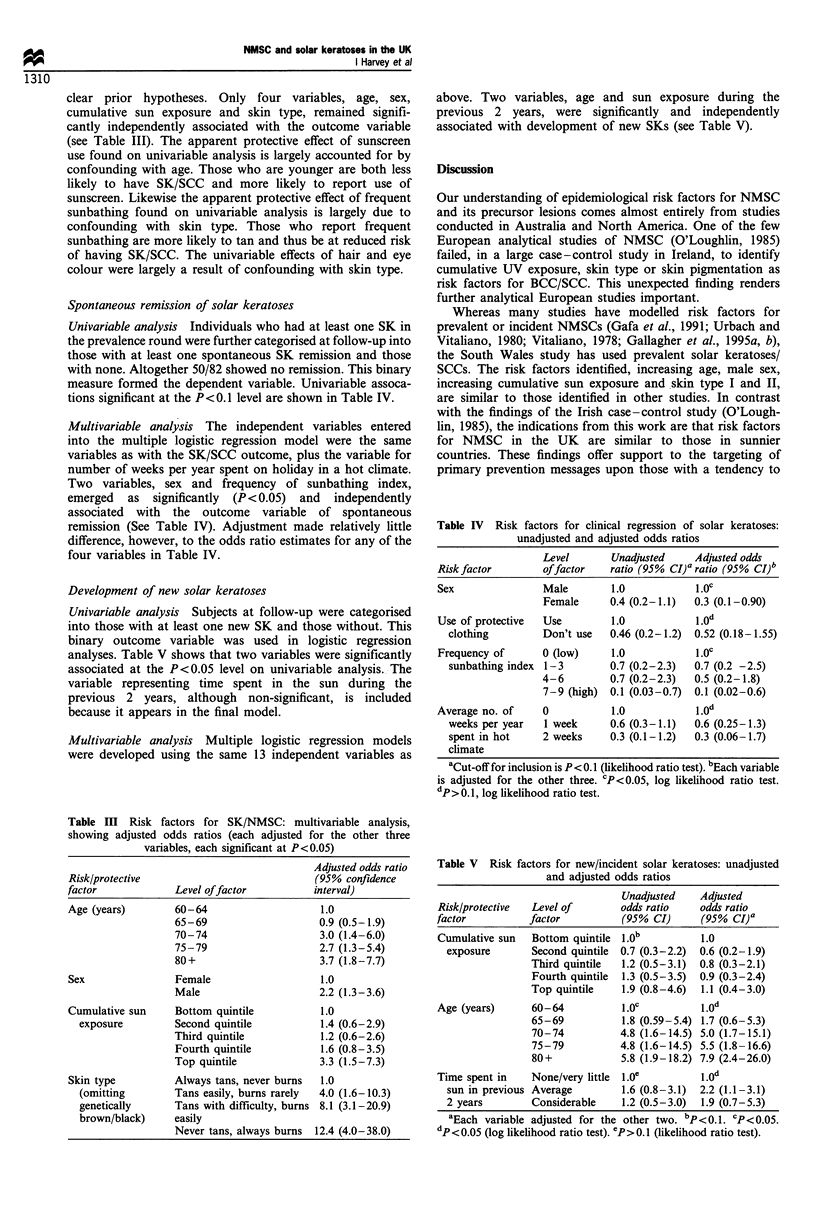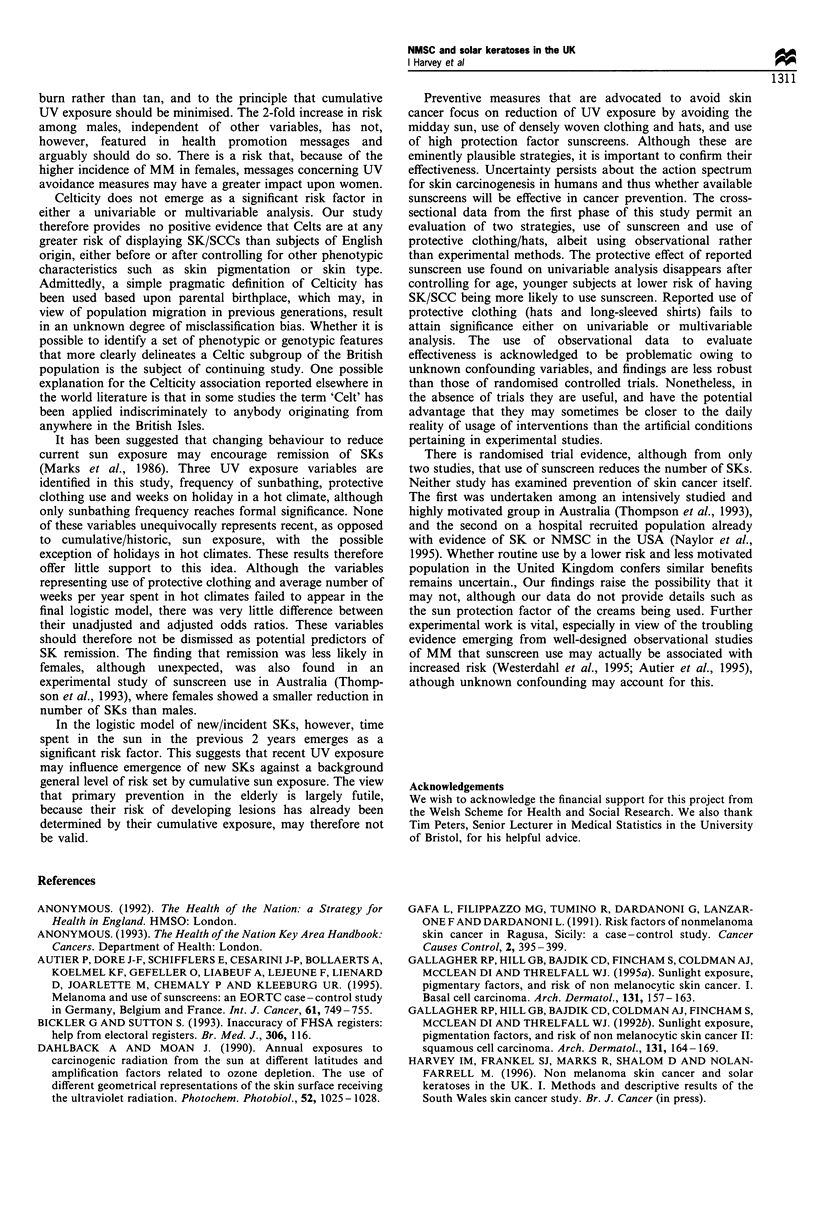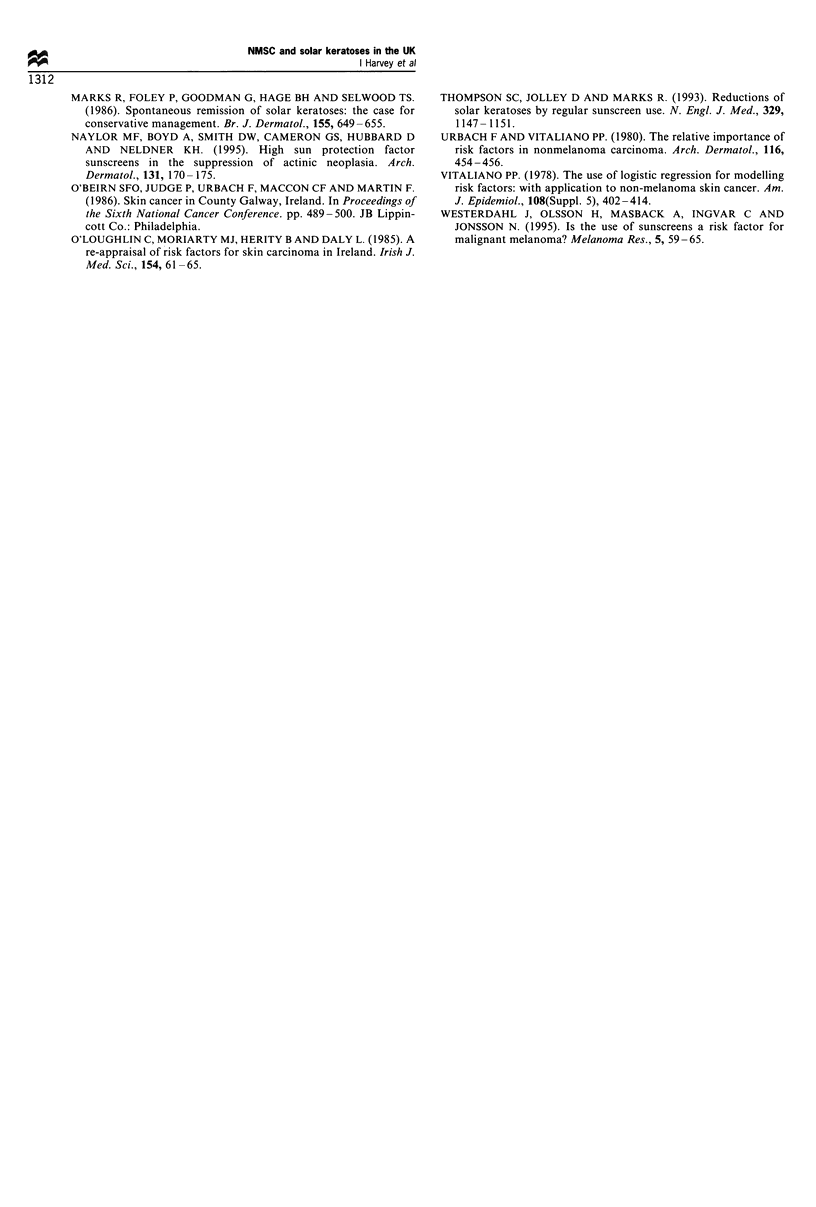# Non-melanoma skin cancer and solar keratoses II analytical results of the South Wales Skin Cancer Study.

**DOI:** 10.1038/bjc.1996.535

**Published:** 1996-10

**Authors:** I. Harvey, S. Frankel, R. Marks, D. Shalom, M. Nolan-Farrell

**Affiliations:** Department of Social Medicine, University of Bristol, UK.

## Abstract

This study aimed to identify risk markers for prevalent solar keratoses (SKs) and squamous cell carcinomata (SCC) combined, for incident SKs and for spontaneous remission of SKs and to evaluate primary preventative measures. It was a cross-sectional study, with follow-up, conducted in South Wales, and involved 1034 subjects aged 60 years and over. The main outcome measures were the presence of and changes in SKs, and presence of skin cancers, on sun-exposed skin, and risk factors for prevalent SKs/SCCs and for incidence and remission of SKs. We found that variables independently associated with prevalent SKs/SCCs were: age [80 + years vs 60-64 years, odds ratio (OR) 3.7]; sex (male vs female OR 2.2); cumulative sun exposure (top quintile vs bottom quintile OR 3.3) and skin type (skin type 1 vs 4 OR 12.4). Use of sunscreen or protective clothing was not protective after controlling for confounders. Males and those who sunbathe infrequently showed greater remission of SKs. Older subjects and those spending most time in the sun in the preceeding 2 years were most likely to develop new SKs. We conclude that the risk factors identified are consistent with results from sunnier countries. The failure of sunscreen or clothing to emerge as protective raises doubts as to whether these measures are as effective in routine use in the general population as theoretical considerations and the limited trial evidence would predict. Recently reported sun exposure appears to influence the risk of developing new SKs.


British Journal of Cancer (1996) 74, 1308-1312
© 1996 Stockton Press All rights reserved 0007-0920/96 $12.00

### Non-melanoma skin cancer and solar keratoses II analytical results of the South Wales skin cancer study

I Harvey[1], S Frankel[1], R Marks[2], D Shalom[2] and M Nolan-Farrell[3]

[1]*Department of Social Medicine, University of Bristol, Canynge Hall, Whiteladies Rd, Bristol BS8 2PR;* [2]*University of Wales College of Medicine, Heath Park, Cardiff CF4 4DH;* [3]*South Glamorgan Health Authority, Temple of Peace and Health, Cathays Park, Cardiff CF1 3XN, UK.*

**Summary** This study aimed to identify risk markers for prevalent solar keratoses (SKs) and squamous cell carcinomata (SCC) combined, for incident SKs and for spontaneous remission of SKs and to evaluate primary preventative measures. It was a cross-sectional study, with follow-up, conducted in South Wales, and involved 1034 subjects aged 60 years and over. The main outcome measures were the presence of and changes in SKs, and presence of skin cancers, on sun-exposed skin, and risk factors for prevalent SKs/SCCs and for incidence and remission of SKs. We found that variables independently associated with prevalent SKs/SCCs were: age [80+ years *vs* 60-64 years, odds ratio (OR) 3.7]; sex (male *vs* female OR 2.2); cumulative sun exposure (top quintile *vs* bottom quintile OR 3.3) and skin type (skin type 1 *vs* 4 OR 12.4). Use of sunscreen or protective clothing was not protective after controlling for confounders. Males and those who sunbathe infrequently showed greater remission of SKs. Older subjects and those spending most time in the sun in the preceeding 2 years were most likely to develop new SKs. We conclude that the risk factors identified are consistent with results from sunnier countries. The failure of sunscreen or clothing to emerge as protective raises doubts as to whether these measures are as effective in routine use in the general population as theoretical considerations and the limited trial evidence would predict. Recently reported sun exposure appears to influence the risk of developing new SKs.

**Keywords:** non-melanoma skin cancer; solar keratoses; epidemiology; risk factor; ultraviolet light; multiple logistic regression

Skin cancer is currently the subject of strong interest in Britain because of its rising incidence and apparently high potential for prevention (Anonymous, 1992; Anonymous, 1993), augmented by concern about the anticipated health effects of the thinning of the ozone layer. A 1% stratospheric ozone depletion should in theory lead to a 1-3% increase in both non-melanoma and melanoma skin cancers (Dahlback and Moan, 1990).

Analytical epidemiological studies especially of non-melanoma skin cancers (NMSCs) and solar keratoses (SKs) in random population samples provide a means, therefore, of identifying and confirming hypothesised epidemiological risk factors and of evaluating, using observational methods, currently recommended preventive measures.

A longitudinal study, of SKs and NMSCs was conducted in South Glamorgan in South Wales for which primary data were gathered between 1988 and 1992. A full introduction to previous research findings concerning skin cancer and to the present study is contained in a companion paper (Harvey *et al.*, 1996).

### Methods

The methods have been described in detail previously (Harvey *et al.*, 1996). Briefly, a random sample of 1034 subjects age 60 years and over was drawn from the South Glamorgan Family Health Services Authority (FHSA) register, and after seeking informed consent, these subjects were visited in their homes by a research registrar in dermatology. A further visit was made 1-2 years later.

At both visits a detailed administered questionnaire was completed and an examination made of the skin of the head and neck, arms (to the shoulder), legs (below the knee) and feet. Polaroid photographs and 35 mm slides were taken during the first visit of suspected solar keratoses and skin cancers. The slides were later used to validate diagnoses and the polaroid photographs to aid in the assessment of previously noted solar keratoses at the follow-up visit. Information about skin type, hair colour at age 15 years and estimated cumulative UV exposure was obtained on both visits in order to examine the test-retest reliability of subjects' responses. Pathological confirmation of suspected skin cancers was obtained wherever available.

Data were coded, entered and analysed using SPSS for Windows version 6.

### *Variables and variable definitions*

Independent variables considered in the data analysis fall into several categories: demographic variables (age, sex, social class); geno/phenotypic variables (skin type, skin pigmentation, hair colour, eye colour, Celticity); UV exposure variables (cumulative UV exposure, sun exposure in the previous 2 years, sunburn in the previous 2 years, frequency of sunbathing index); behavioural variables [use of sunscreen, use of protective clothing (hat and long sleeves), use of UV lamps/sunbeds]. These variables were selected in part from knowledge of epidemiological studies conducted elsewhere in the world, and in part to evaluate, using observational methods, the effectiveness of certain plausible preventive measures, such as use of sunscreen and protective clothing, that are currently widely recommended.

The definitions of key independent variables are shown in Table I.

### *Sample size*

The measure used to determine the sample size was an estimated prevalence of solar keratoses. Assuming a prevalence of 10.6% (as found in a previous study in Ireland, O'Beirn *et al.*, 1968), and aiming to have 95% confidence limits around this point estimate no wider than ±2.5%, required 580 subjects. Allowing for 20% non-response and a further 20% for FHSA register inaccuracy

Correspondence: I Harvey
Received 11 December 1995; revised 7 May 1996; accepted 14 May 1996

Table I Definitions of important independent variables

| Variable name | Variable definition |
|---|---|
| Skin type | Reaction of skin when first exposed to the sun for 1–2 h unprotected at the start of the summer, using Fitzpatrick classification (never tans, always burns to always tans, never burns) |
| Cumulative sun exposure | Lifetime number of hours UV exposure, based upon estimated hours of outdoor exposure (weekdays and weekend days considered separately) for ages 20–39 years; 40–59 years; 60 years+ |
| Sun exposure in the previous two years | Self-assessed on four point scale from none to considerable |
| Skin pigmentation | Assessed on four point scale from skin of upper inner arm |
| Frequency of sunbathing index | Unweighted sum of scores for self-reported sunbathing frequency (never, 0; rarely, 1; occasionally, 2; frequently, 3; summated across three life periods (0–39, 40–59, 60+ years) |
| Sunscreen use | Response to question 'do you normally use sunscreen when in the sun?' |
| Protective clothing | Response to question 'do you normally wear protective clothing (long sleeves, hat) when in the sun?' |
| UV lamp/sunbed use | Categorised into never *vs* ever used |
| Celticity index | Index scoring 2 for each parent born in a Celtic country (Wales, Scotland, Ireland) range 0–2 |

(Bickler *et al.*, 1993), a random sample of 1034 subjects was drawn from the FHSA register.

*Statistical methods*

For the univariable analyses, likelihood ratio tests (derived from univariable logistic regression) and chi-square tests for linear trend were used as tests of significance.

In the construction of multivariable logistic regression models an approach was adopted which has the advantage of identifying and allowing the inclusion of variables that may be highly correlated with other independent variables under consideration. Predictor variables were dropped individually from a model containing all those variables under scrutiny. Those exerting a significant independent effect at the $P<0.05$ level (log likelihood ratio test) were then placed in a model to which the remaining (non-significant) predictor variables were added individually. Any of these added variables now attaining significance were incorporated into the final model, and the remaining non-significant variables again added individually in an iterative fashion. This combination of (non-automatic) backward and forward stepwise modelling means that highly correlated variables which may not be identified in the backward steps will be identified during the forward modelling steps. Confidence intervals for unadjusted and adjusted odds ratios were derived from the standard errors of the logistic regression coefficients.

Variables were selected for inclusion in the multiple logistic models partly on the basis of significant association on univariable analysis and partly because of clear prior hypotheses. Lack of significant univariable association did not therefore preclude inclusion in the multivariable model. The failure of a variable to register as significant on univariable analysis can potentially be caused by suppression by other variables, which may become apparent in multivariable analysis. A more liberal cut-off alpha value (0.1) than the conventional 0.05 level was used in the selection of variables when modelling remission of and incidence of solar keratoses. This was because there were exploratory analyses where previous published studies provide little guidance about variable selection.

### Results

Response rates were 70.7% in round 1 and 79.3% in round 2. Non-responders in both rounds were more likely to be older than responders.

*Risk factors for solar keratoses/squamous cell carcinoma*

*Univariable analysis* In this analysis the presence of SKs and SCCs has been amalgamated into a single outcome variable from which subjects with basal cell carcinoma (BCC) alone or malignant melanoma (MM) alone have been omitted. Those variables significantly associated with the outcome variable on univariable analysis and the unadjusted odds ratios associated with each level of each variable are shown in Table II. A number of variables were not significantly associated (at the $P=0.05$ level) with the presence of SK/SCC. These were: social class, sunburn during the previous 2 years, time spent in the sun in the previous 2 years, Celticity, use of UV lamps/sunbeds, and use of protective clothing.

*Multivariable analysis* Thirteen variables were entered into multiple logistic regression models to determine which were independently associated with the presence of SK/SCC after adjustment for the others. These variables were: all the variables (see Table II) which were significantly associated on univariable analysis; four additional variables (UV lamp/sunbed use; sunburn in the previous 2 years; use of protective clothing; Celticity) selected because they were the subject of

Table II Risk/protective factors for solar keratoses/non-melanoma skin cancers: univariable analysis

| Risk/protective factor | Level of factor | Unadjusted odds ratio (95% confidence interval) |
|---|---|---|
| Age (years) | 60–64 | 1.0 |
| | 65–69 | 1.1 (0.5–2.0) |
| | 70–74 | 3.0 (1.6–5.8) |
| | 75–79 | 3.8 (2.0–7.2) |
| | 80+ | 3.9 (2.0–7.4) |
| Sex | Female | 1.0 |
| | Male | 2.4 (1.6–3.5) |
| Cumulative sun exposure | Bottom quintile | 1.0 |
| | Second quintile | 1.3 (0.7–2.7) |
| | Third quintile | 1.3 (0.6–2.6) |
| | Fourth quintile | 1.9 (1.0–3.7) |
| | Top quintile | 4.3 (2.3–8.2) |
| Skin type (omitting genetically brown/black) | Always tans, never burns | 1.0 |
| | Tans easily, burns rarely | 3.0 (1.2–7.4) |
| | Tans with difficulty, burns easily | 5.3 (2.2–12.7) |
| | Never tans, always burns | 8.0 (2.9–22.1) |
| Hair colour | Black | 1.0 |
| | Dark brown | 1.0 (0.4–2.2) |
| | Medium brown | 1.1 (0.5–2.5) |
| | Light brown | 1.5 (0.6–3.4) |
| | Red/blonde | 1.7 (0.7–4.2)** |
| Skin pigmentation | Dark | 1.0 |
| | Medium | 5.1 (0.7–38.5) |
| | Fair | 10.9 (1.4–82.0) |
| Frequency of sunbathing index | 0 (low) | 1.0 |
| | 1–3 | 0.8 (0.5–1.4) |
| | 4–6 | 0.6 (0.4–1.0) |
| | 7–9 (high) | 0.5 (0.3–0.8) |
| Use of sunscreen | Yes | 1.0 |
| | No | 1.8 (1.2–2.9) |
| Eye colour | Blue and green | 1.0 |
| | Brown and grey | 0.6 (0.4–1.0) |

**Chi-square test for linear trend, $P<0.05$. Other variables, likelihood ratio test, $P<0.05$.

clear prior hypotheses. Only four variables, age, sex, cumulative sun exposure and skin type, remained significantly independently associated with the outcome variable (see Table III). The apparent protective effect of sunscreen use found on univariable analysis is largely accounted for by confounding with age. Those who are younger are both less likely to have SK/SCC and more likely to report use of sunscreen. Likewise the apparent protective effect of frequent sunbathing found on univariable analysis is largely due to confounding with skin type. Those who report frequent sunbathing are more likely to tan and thus be at reduced risk of having SK/SCC. The univariable effects of hair and eye colour were largely a result of confounding with skin type.

*Spontaneous remission of solar keratoses*

*Univariable analysis* Individuals who had at least one SK in the prevalence round were further categorised at follow-up into those with at least one spontaneous SK remission and those with none. Altogether 50/82 showed no remission. This binary measure formed the dependent variable. Univariable assocations significant at the $P<0.1$ level are shown in Table IV.

*Multivariable analysis* The independent variables entered into the multiple logistic regression model were the same variables as with the SK/SCC outcome, plus the variable for number of weeks per year spent on holiday in a hot climate. Two variables, sex and frequency of sunbathing index, emerged as significantly ($P<0.05$) and independently associated with the outcome variable of spontaneous remission (See Table IV). Adjustment made relatively little difference, however, to the odds ratio estimates for any of the four variables in Table IV.

*Development of new solar keratoses*

*Univariable analysis* Subjects at follow-up were categorised into those with at least one new SK and those without. This binary outcome variable was used in logistic regression analyses. Table V shows that two variables were significantly associated at the $P<0.05$ level on univariable analysis. The variable representing time spent in the sun during the previous 2 years, although non-significant, is included because it appears in the final model.

*Multivariable analysis* Multiple logistic regression models were developed using the same 13 independent variables as above. Two variables, age and sun exposure during the previous 2 years, were significantly and independently associated with development of new SKs (see Table V).

**Table III** Risk factors for SK/NMSC: multivariable analysis, showing adjusted odds ratios (each adjusted for the other three variables, each significant at $P<0.05$)

| *Risk/protective factor* | *Level of factor* | *Adjusted odds ratio (95% confidence interval)* |
|---|---|---|
| Age (years) | 60–64 | 1.0 |
| | 65–69 | 0.9 (0.5–1.9) |
| | 70–74 | 3.0 (1.4–6.0) |
| | 75–79 | 2.7 (1.3–5.4) |
| | 80+ | 3.7 (1.8–7.7) |
| Sex | Female | 1.0 |
| | Male | 2.2 (1.3–3.6) |
| Cumulative sun exposure | Bottom quintile | 1.0 |
| | Second quintile | 1.4 (0.6–2.9) |
| | Third quintile | 1.2 (0.6–2.6) |
| | Fourth quintile | 1.6 (0.8–3.5) |
| | Top quintile | 3.3 (1.5–7.3) |
| Skin type (omitting genetically brown/black) | Always tans, never burns | 1.0 |
| | Tans easily, burns rarely | 4.0 (1.6–10.3) |
| | Tans with difficulty, burns easily | 8.1 (3.1–20.9) |
| | Never tans, always burns | 12.4 (4.0–38.0) |

### Discussion

Our understanding of epidemiological risk factors for NMSC and its precursor lesions comes almost entirely from studies conducted in Australia and North America. One of the few European analytical studies of NMSC (O'Loughlin, 1985) failed, in a large case–control study in Ireland, to identify cumulative UV exposure, skin type or skin pigmentation as risk factors for BCC/SCC. This unexpected finding renders further analytical European studies important.

Whereas many studies have modelled risk factors for prevalent or incident NMSCs (Gafa *et al.*, 1991; Urbach and Vitaliano, 1980; Vitaliano, 1978; Gallagher *et al.*, 1995*a*, *b*), the South Wales study has used prevalent solar keratoses/SCCs. The risk factors identified, increasing age, male sex, increasing cumulative sun exposure and skin type I and II, are similar to those identified in other studies. In contrast with the findings of the Irish case–control study (O'Loughlin, 1985), the indications from this work are that risk factors for NMSC in the UK are similar to those in sunnier countries. These findings offer support to the targeting of primary prevention messages upon those with a tendency to

**Table IV** Risk factors for clinical regression of solar keratoses: unadjusted and adjusted odds ratios

| *Risk factor* | *Level of factor* | *Unadjusted ratio (95% CI)*[a] | *Adjusted odds ratio (95% CI)*[b] |
|---|---|---|---|
| Sex | Male | 1.0 | 1.0[c] |
| | Female | 0.4 (0.2–1.1) | 0.3 (0.1–0.90) |
| Use of protective clothing | Use | 1.0 | 1.0[d] |
| | Don't use | 0.46 (0.2–1.2) | 0.52 (0.18–1.55) |
| Frequency of sunbathing index | 0 (low) | 1.0 | 1.0[c] |
| | 1–3 | 0.7 (0.2–2.3) | 0.7 (0.2 –2.5) |
| | 4–6 | 0.7 (0.2–2.3) | 0.5 (0.2–1.8) |
| | 7–9 (high) | 0.1 (0.03–0.7) | 0.1 (0.02–0.6) |
| Average no. of weeks per year spent in hot climate | 0 | 1.0 | 1.0[d] |
| | 1 week | 0.6 (0.3–1.1) | 0.6 (0.25–1.3) |
| | 2 weeks | 0.3 (0.1–1.2) | 0.3 (0.06–1.7) |

[a]Cut-off for inclusion is $P<0.1$ (likelihood ratio test). [b]Each variable is adjusted for the other three. [c]$P<0.05$, log likelihood ratio test. [d]$P>0.1$, log likelihood ratio test.

**Table V** Risk factors for new/incident solar keratoses: unadjusted and adjusted odds ratios

| *Risk/protective factor* | *Level of factor* | *Unadjusted odds ratio (95% CI)* | *Adjusted odds ratio (95% CI)*[a] |
|---|---|---|---|
| Cumulative sun exposure | Bottom quintile | 1.0[b] | 1.0 |
| | Second quintile | 0.7 (0.3–2.2) | 0.6 (0.2–1.9) |
| | Third quintile | 1.2 (0.5–3.1) | 0.8 (0.3–2.1) |
| | Fourth quintile | 1.3 (0.5–3.5) | 0.9 (0.3–2.4) |
| | Top quintile | 1.9 (0.8–4.6) | 1.1 (0.4–3.0) |
| Age (years) | 60–64 | 1.0[c] | 1.0[d] |
| | 65–69 | 1.8 (0.59–5.4) | 1.7 (0.6–5.3) |
| | 70–74 | 4.8 (1.6–14.5) | 5.0 (1.7–15.1) |
| | 75–79 | 4.8 (1.6–14.5) | 5.5 (1.8–16.6) |
| | 80+ | 5.8 (1.9–18.2) | 7.9 (2.4–26.0) |
| Time spent in sun in previous 2 years | None/very little | 1.0[e] | 1.0[d] |
| | Average | 1.6 (0.8–3.1) | 2.2 (1.1–3.1) |
| | Considerable | 1.2 (0.5–3.0) | 1.9 (0.7–5.3) |

[a]Each variable adjusted for the other two. [b]$P<0.1$. [c]$P<0.05$. [d]$P<0.05$ (log likelihood ratio test). [e]$P>0.1$ (likelihood ratio test).

burn rather than tan, and to the principle that cumulative UV exposure should be minimised. The 2-fold increase in risk among males, independent of other variables, has not, however, featured in health promotion messages and arguably should do so. There is a risk that, because of the higher incidence of MM in females, messages concerning UV avoidance measures may have a greater impact upon women.

Celticity does not emerge as a significant risk factor in either a univariable or multivariable analysis. Our study therefore provides no positive evidence that Celts are at any greater risk of displaying SK/SCCs than subjects of English origin, either before or after controlling for other phenotypic characteristics such as skin pigmentation or skin type. Admittedly, a simple pragmatic definition of Celticity has been used based upon parental birthplace, which may, in view of population migration in previous generations, result in an unknown degree of misclassification bias. Whether it is possible to identify a set of phenotypic or genotypic features that more clearly delineates a Celtic subgroup of the British population is the subject of continuing study. One possible explanation for the Celticity association reported elsewhere in the world literature is that in some studies the term 'Celt' has been applied indiscriminately to anybody originating from anywhere in the British Isles.

It has been suggested that changing behaviour to reduce current sun exposure may encourage remission of SKs (Marks *et al*., 1986). Three UV exposure variables are identified in this study, frequency of sunbathing, protective clothing use and weeks on holiday in a hot climate, although only sunbathing frequency reaches formal significance. None of these variables unequivocally represents recent, as opposed to cumulative/historic, sun exposure, with the possible exception of holidays in hot climates. These results therefore offer little support to this idea. Although the variables representing use of protective clothing and average number of weeks per year spent in hot climates failed to appear in the final logistic model, there was very little difference between their unadjusted and adjusted odds ratios. These variables should therefore not be dismissed as potential predictors of SK remission. The finding that remission was less likely in females, although unexpected, was also found in an experimental study of sunscreen use in Australia (Thompson *et al*., 1993), where females showed a smaller reduction in number of SKs than males.

In the logistic model of new/incident SKs, however, time spent in the sun in the previous 2 years emerges as a significant risk factor. This suggests that recent UV exposure may influence emergence of new SKs against a background general level of risk set by cumulative sun exposure. The view that primary prevention in the elderly is largely futile, because their risk of developing lesions has already been determined by their cumulative exposure, may therefore not be valid.

Preventive measures that are advocated to avoid skin cancer focus on reduction of UV exposure by avoiding the midday sun, use of densely woven clothing and hats, and use of high protection factor sunscreens. Although these are eminently plausible strategies, it is important to confirm their effectiveness. Uncertainty persists about the action spectrum for skin carcinogenesis in humans and thus whether available sunscreens will be effective in cancer prevention. The cross-sectional data from the first phase of this study permit an evaluation of two strategies, use of sunscreen and use of protective clothing/hats, albeit using observational rather than experimental methods. The protective effect of reported sunscreen use found on univariable analysis disappears after controlling for age, younger subjects at lower risk of having SK/SCC being more likely to use sunscreen. Reported use of protective clothing (hats and long-sleeved shirts) fails to attain significance either on univariable or multivariable analysis. The use of observational data to evaluate effectiveness is acknowledged to be problematic owing to unknown confounding variables, and findings are less robust than those of randomised controlled trials. Nonetheless, in the absence of trials they are useful, and have the potential advantage that they may sometimes be closer to the daily reality of usage of interventions than the artificial conditions pertaining in experimental studies.

There is randomised trial evidence, although from only two studies, that use of sunscreen reduces the number of SKs. Neither study has examined prevention of skin cancer itself. The first was undertaken among an intensively studied and highly motivated group in Australia (Thompson *et al*., 1993), and the second on a hospital recruited population already with evidence of SK or NMSC in the USA (Naylor *et al*., 1995). Whether routine use by a lower risk and less motivated population in the United Kingdom confers similar benefits remains uncertain., Our findings raise the possibility that it may not, although our data do not provide details such as the sun protection factor of the creams being used. Further experimental work is vital, especially in view of the troubling evidence emerging from well-designed observational studies of MM that sunscreen use may actually be associated with increased risk (Westerdahl *et al*., 1995; Autier *et al*., 1995), athough unknown confounding may account for this.

**Acknowledgements**
We wish to acknowledge the financial support for this project from the Welsh Scheme for Health and Social Research. We also thank Tim Peters, Senior Lecturer in Medical Statistics in the University of Bristol, for his helpful advice.

### References

ANONYMOUS. (1992). *The Health of the Nation: a Strategy for Health in England.* HMSO: London.

ANONYMOUS. (1993). *The Health of the Nation Key Area Handbook: Cancers.* Department of Health: London.

AUTIER P, DORE J-F, SCHIFFLERS E, CESARINI J-P, BOLLAERTS A, KOELMEL KF, GEFELLER O, LIABEUF A, LEJEUNE F, LIENARD D, JOARLETTE M, CHEMALY P AND KLEEBURG UR. (1995). Melanoma and use of sunscreens: an EORTC case–control study in Germany, Belgium and France. *Int. J. Cancer*, **61**, 749–755.

BICKLER G AND SUTTON S. (1993). Inaccuracy of FHSA registers: help from electoral registers. *Br. Med. J.*, **306**, 116.

DAHLBACK A AND MOAN J. (1990). Annual exposures to carcinogenic radiation from the sun at different latitudes and amplification factors related to ozone depletion. The use of different geometrical representations of the skin surface receiving the ultraviolet radiation. *Photochem. Photobiol.*, **52**, 1025–1028.

GAFA L, FILIPPAZZO MG, TUMINO R, DARDANONI G, LANZARONE F AND DARDANONI L. (1991). Risk factors of nonmelanoma skin cancer in Ragusa, Sicily: a case–control study. *Cancer Causes Control*, **2**, 395–399.

GALLAGHER RP, HILL GB, BAJDIK CD, FINCHAM S, COLDMAN AJ, MCCLEAN DI AND THRELFALL WJ. (1995*a*). Sunlight exposure, pigmentary factors, and risk of non melanocytic skin cancer. I. Basal cell carcinoma. *Arch. Dermatol.*, **131**, 157–163.

GALLAGHER RP, HILL GB, BAJDIK CD, COLDMAN AJ, FINCHAM S, MCCLEAN DI AND THRELFALL WJ. (1992*b*). Sunlight exposure, pigmentation factors, and risk of non melanocytic skin cancer II: squamous cell carcinoma. *Arch. Dermatol.*, **131**, 164–169.

HARVEY IM, FRANKEL SJ, MARKS R, SHALOM D AND NOLAN-FARRELL M. (1996). Non melanoma skin cancer and solar keratoses in the UK. I. Methods and descriptive results of the South Wales skin cancer study. *Br. J. Cancer* (in press).

MARKS R, FOLEY P, GOODMAN G, HAGE BH AND SELWOOD TS. (1986). Spontaneous remission of solar keratoses: the case for conservative management. *Br. J. Dermatol.*, **155,** 649–655.

NAYLOR MF, BOYD A, SMITH DW, CAMERON GS, HUBBARD D AND NELDNER KH. (1995). High sun protection factor sunscreens in the suppression of actinic neoplasia. *Arch. Dermatol.*, **131,** 170–175.

O'BEIRN SFO, JUDGE P, URBACH F, MACCON CF AND MARTIN F. (1986). Skin cancer in County Galway, Ireland. In *Proceedings of the Sixth National Cancer Conference*. pp. 489–500. JB Lippincott Co.: Philadelphia.

O'LOUGHLIN C, MORIARTY MJ, HERITY B AND DALY L. (1985). A re-appraisal of risk factors for skin carcinoma in Ireland. *Irish J. Med. Sci.*, **154,** 61–65.

THOMPSON SC, JOLLEY D AND MARKS R. (1993). Reductions of solar keratoses by regular sunscreen use. *N. Engl. J. Med.*, **329,** 1147–1151.

URBACH F AND VITALIANO PP. (1980). The relative importance of risk factors in nonmelanoma carcinoma. *Arch. Dermatol.*, **116,** 454–456.

VITALIANO PP. (1978). The use of logistic regression for modelling risk factors: with application to non-melanoma skin cancer. *Am. J. Epidemiol.*, **108**(Suppl. 5), 402–414.

WESTERDAHL J, OLSSON H, MASBACK A, INGVAR C AND JONSSON N. (1995). Is the use of sunscreens a risk factor for malignant melanoma? *Melanoma Res.*, **5,** 59–65.